# Clinicopathologic comparison of basal cell carcinoma among a diverse patient population in Los Angeles County

**DOI:** 10.1002/ski2.379

**Published:** 2024-03-24

**Authors:** Esther Choi, Martha A. Oberg, Maya Hijazi, Luke Hall, Kimberly A. Miller, Arjun Mehta, Stephen Capone, Gino K. In

**Affiliations:** ^1^ Elson S. Floyd College of Medicine Washington State University Spokane Washington USA; ^2^ Department of Pathology Los Angeles County ‐ University of Southern California Medical Center Los Angeles California USA; ^3^ Department of Population and Public Health Sciences Keck School of Medicine University of Southern California Los Angeles California USA; ^4^ Department of Dermatology Keck School of Medicine University of Southern California Los Angeles California USA; ^5^ Department of Neurology Virginia Tech University Roanoke Virginia USA; ^6^ Division of Medical Oncology Norris Comprehensive Cancer Center University of Southern California Los Angeles California USA

## Abstract

**Introduction:**

Basal cell carcinoma (BCC) is the most common malignancy in the United States. The majority of cases are identified in Non‐Hispanic Whites (NHW) and are far less demonstrated in patients of colour (POC). However, the Hispanic population represents a large and growing proportion of the US population, and skin cancer diagnoses in Hispanics are rising. Thus, the goal of this study is to examine clinicopathologic differences between BCC in Hispanics versus NHW.

**Methods:**

A retrospective chart review of Hispanic and NHW patients with BCC at Los Angeles County + USC Medical Center from January 2018 to March 2020 was performed. In total, 101 BCC samples from the first 100 patients identified of Hispanic ancestry, as well as 50 BCC samples identified from the first 50 patients identifying as NHW, were included for analysis. Patient characteristics (age, sex, medical history, and ethnicity), as well as tumour characteristics (location, subtype, tumour depth, and perineural invasion), were collected. We used between subjects *t*‐tests for continuous variables, and chi‐square tests for categorical variables.

**Results:**

In total, 151 specimens were collected amongst 122 subjects (79 Hispanics and 43 NHW patients). Among NHW, the majority of patients (74.4%) were men, but among the Hispanic population, the majority (68.4%) were female (*p* < 0.001). Prior history of other skin cancer was more common among NHW (67.4%) than Hispanics (31.6%) (*p*=<0.001). The Hispanic population had a significantly higher proportion of head and neck tumours (*p* = 0.0004) but a lower proportion of extremity tumours (*p* = 0.001) compared to NHW. Pigmented BCC was significantly more common among Hispanic patients (*p* < 0.01). Finally, within the Hispanic group, there was a significant association between sex and histology (*p* = 0.004), with Hispanic men demonstrating more aggressive mix histology compared to Hispanic women.

**Discussion:**

Our study supports the notion that BCC disparities occur among POC compared to NHW. This includes variations in epidemiologic factors such as sex and past medical history, primary tumour location, and pathologic characteristics. Further research should be conducted to identify additional differences in skin cancer presentation in POC to reduce the gaps in skin cancer knowledge and care.

## Graphical abstract

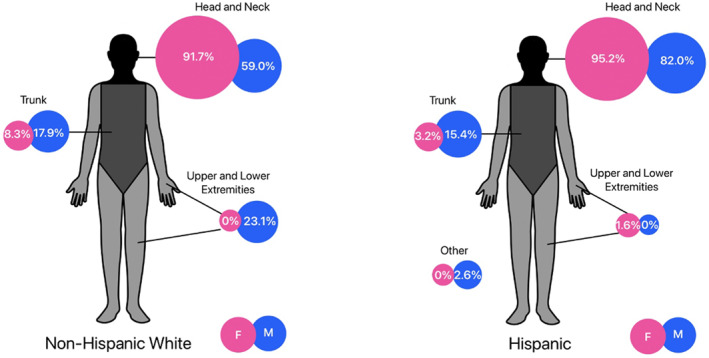

**What is already known about this topic?**

- Basal cell carcinomas are the most common skin cancer in the Hispanic population.
- Available studies suggest that there are worse outcomes and higher morbidity in Hispanic patients with basal cell carcinoma (BCC) compared to Non‐Hispanic White patients.

**What does this study add?**

- Our study demonstrates that differences exist in the clinicopathologic presentation of BCC amongst Hispanic populations, including differences in sex, primary tumour location, and pathologic characteristics.
- This information will be impertinent in caring for the Hispanic population.

## 1 INTRODUCTION

Basal cell carcinoma (BCC) is the most common malignancy in the United States, with an estimated 3.6 million new cases diagnosed each year.^1^ However, a lack of standardised reporting of BCC to tumour registries may grossly underestimate the true burden of disease.^1^, ^2^, ^3^, ^4^, ^5^ Most studies of BCC have been restricted to North America, Europe and Australia, where BCC is more common and representative of a more homogenous, non‐Hispanic White (NHW) patient population. As a result, there is relatively limited understanding of the impact of BCC among patients of colour (POC), including Hispanic, Black and Asian populations.^6^, ^7^ In a large BCC registry from an integrated healthcare system, POC constituted 8% of the total BCC patient population studied, with Hispanics attributing 3.1% of this total, Asians 1%, and Blacks 0.2%.^8^ While overall rates of BCC may be lower among POC, a growing number of studies suggest worse outcomes, such as surgical defect size and post‐treatment morbidity, for POC with BCC and other skin cancers compared to NHW.^9^, ^10^, ^11^, ^12^ These disparities may be driven by a number of complex and contributing factors, such as low awareness, poor use of sun protection behaviour, delays in diagnosis, advanced stages at presentation, and atypical clinicopathologic presentations of BCC.^2^, ^6^, ^13^, ^14^, ^15^, ^16^, ^17^ According to the U.S Census Bureau, the Hispanic population is the largest racio‐ethnic group in the US, comprising 18.7% of the total US population.^18^ It has been estimated that BCC accounts for nearly 50%–60% of all cutaneous malignancies among the Hispanic population.^13^, ^19^ As BCC and other skin cancers are increasing in incidence among all racial and ethnic groups, there is an urgent need to describe the features of BCC among patients of Hispanic ancestry.^19^, ^20^, ^21^, ^22^, ^23^ The goal of this study is to examine the presentation and histopathology of BCC at a diverse safety net hospital in Los Angeles County serving a predominantly Hispanic patient population with the hopes of addressing gaps in skin cancer knowledge for this group.

## 2 METHODS

A retrospective chart review of patients with BCC treated at the Los Angeles County + University of Southern California Medical Center was conducted between January 2018 and March 2020. To facilitate adequate sample size to allow for comparison across different racial/ethnic groups, the first 100 BCC samples identified from patients of Hispanic ancestry, as well as the first 50 BCC samples identified from NHW, were included for analysis. Patients who did not have histologic confirmation of BCC, or who did not have corresponding clinical information available from medical records, were excluded. This study was approved by the Institutional Review Board at the University of Southern California. Patient demographics, including age, sex, ethnicity, comorbidities, prior history of other skin cancers and prior history of other non‐skin cancers were collected. Tumour characteristics, including anatomic tumour location, tumour thickness, histologic subtype, pigmentation, perineural invasion and other pathological features were reported. Patients with multiple BCC were counted as one subject when evaluating age and past medical history but counted as distinct BCC cases when evaluating for anatomic location and other tumour characteristics. Histological subtypes were categorised as superficial, nodular, indeterminate, aggressive mix, and non‐aggressive mix. Aggressive mix was identified as the presence of infiltrating types. Non‐aggressive mix included the presence of both nodular and superficial features, or infundibulocystic type. Histology subtype was verified for all specimens by a board‐certified pathologist. Descriptive statistics were calculated for all patients, as well as clinicopathological features. Means and standard deviations were computed for continuous variables, while frequencies and percentages were computed for categorical variables. In order to test if participants varied across racial/ethnic groups, we used between subjects *t*‐tests for continuous variables. Chi‐square tests were used for categorical variables to test for associations between race/ethnicity and tumour characteristics. When more than 20% of the expected counts were less than 5, Fisher's exact test was used. If any of the overall tests were statistically significant at the *p* < 0.05 level of significance, we performed a post‐hoc analysis to examine pair‐wise differences. To account for multiple comparisons, the *p*‐value was corrected using the Bonferroni adjustment method. All statistical analyses were performed using R Statistical software (Version 4.2.3; R Foundation for Statistical Computing, Vienna, Australia).

## 3 RESULTS

### 3.1 Patient characteristics

In total, 151 BCC specimens were collected amongst 122 unique subjects. Among these 122 subjects, there were 43 NHW patients (35.2%), from which there were a total of 51 (33.6%) BCC specimens, and 79 (64.8%) Hispanic patients, entailing 101 (66.4%) BCC specimens. Among the NHW patients with BCC, the majority (74.4%) were males, while among the Hispanic BCC patients, the majority (68.4%) were females (*p* < 0.001). The median age at diagnosis for the entire sample was 63 (range 29–96), with the median age 63 years (range 29–80) for NHW compared to 64 years (34–96 years) for Hispanics (*p* = 0.27). Among NHW, 67.4% had a prior history of skin cancer, compared to 31.6% of Hispanics (*p*=<0.001). The proportion of NHW patients with a prior history of other non‐skin cancer was 14.0%, compared to 11.4% among Hispanics (*p* = 0.90).

### 3.2 Tumour characteristics

**FIGURE 1:**
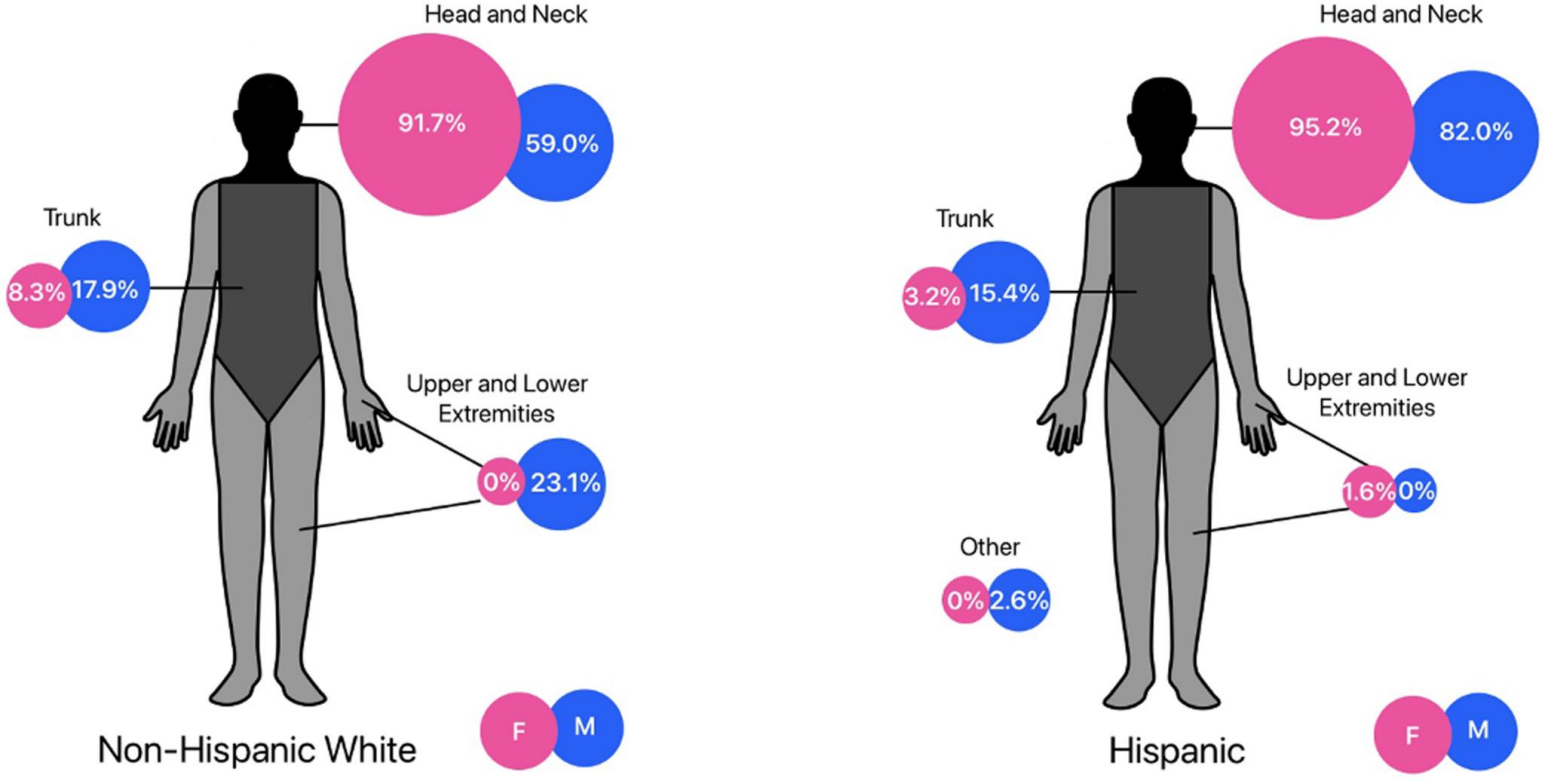
Anatomic distribution of basal cell carcinoma in males compared to females in the Hispanic and Non‐Hispanic White population.

Tumour characteristics are reported in Table 1. Among NHW, the most common anatomic site of tumour involvement was the head/neck region, accounting for 66.7% of all BCC; this was followed by the extremities (17.6%), then trunk (15.7%). Among Hispanics, the most common location for BCC was also the head/neck (90.1%), then followed by trunk (7.9%), and extremities (1.0%); there was one ‘other category’, being located on the scrotum (1.0%). When comparing the tumour location across racioethnic groups (Figure 1), there was a significantly higher proportion of Hispanics with head/neck tumours (66.7% in NHW vs. 90.1% in Hispanics, *p* = 0.0004), and a significantly lower proportion of Hispanics with BCC on the extremities (17.7% of NHW compared to <1% of Hispanics, *p* = 0.001). The proportion of NHW with involvement of the trunk was numerically higher than that for Hispanics but not statistically significant (15.7% in NHW vs. 6.8% in Hispanics, *p* = 0.08). The laterality of tumour location was similar for both populations. Histologically, most tumours were thin (<1 mm) for both NHW (56.8%) and Hispanic (58.4%) patients. The most common histology subtype for both NHW and Hispanics, respectively, was nodular (49.0%, 64.4%), followed by aggressive mix (27.5%, 16.8%), and superficial (15.7%, 7.9%). However, when comparing the histological subtypes between both groups, there was no statistically significant difference (*p* = 0.4). The proportion of pigmented BCC lesions was significantly more prevalent among Hispanics compared to NHW (58.4% vs. 15.7%, respectively, *p*=<0.01).

**TABLE 1:** Characteristics of BCC Tumour Specimens, by Race. Anatomic distribution and histologic characteristics of basal cell carcinoma lesions among Non‐Hispanic Whites and Hispanic groups.

	Non‐Hispanic White (%)	Hispanic (%)	*p*‐value (0.05)
Location			
Head/neck	34 (66.7)	91 (90.1)	<0.001
Extremities	9 (17.6)	1 (1.0)	
Trunk	8 (15.7)	8 (7.9)	
Other	0 (0.0)	1 (1.0)	
Laterality			
Right	27 (53.0)	52 (51.5)	0.90
Left	22 (43.1)	42 (41.6)	
Midline	2 (3.9)	7 (6.9)	
Tumour thickness			
<1 mm	29 (56.9)	59 (58.4)	0.50
1–2 mm	17 (33.3)	29 (28.7)	
2–6 mm	4 (7.8)	13 (12.8)	
>6 mm	1 (1.9)	0	
Perineural invasion			
Yes	0 (0.0)	2 (2.0)	0.60
No	51 (100.0)	99 (98.0)	
Subtype			
Superficial	8 (15.7)	8 (7.9)	0.40
Nodular	25 (49.0)	65 (64.4)	
Non‐aggressive mix^a^	3 (5.9)	8 (7.9)	
Aggressive mix^b^	14 (27.5)	17 (16.8)	
Indeterminant	1 (1.9)	3 (3.0)	
Pigmented			
Yes	8 (15.7)	59 (58.4)	<0.001
No	43 (84.3)	42 (41.6)	

^a^ Non‐aggressive mix included the presence of both nodular and superficial features, or infundibulocystic type.

^b^ Aggressive mix included infiltrating subtypes.

### 3.3 Association between clinical variables and tumour characteristics

Statistical testing revealed several associations when comparing both patient‐specific variables and clinicopathological features. Among the NHW patients, results from the Fischer's exact tests revealed a significant relationship between sex and histology (*p* = 0.04), tumour location and histology (*p* = 0.004), as well as thickness and histology (*p* = 0.03); however, when we conducted pairwise comparisons and adjusted *p*‐values to control for type I error rate, there were no significant associations.

Within the Hispanic group, Fischer's exact tests revealed similar outcomes in the comparison of sex to location (*p* = 0.04) but pairwise comparisons revealed no significant associations after adjusting for multiple comparisons. Similarly, a significant association between history of prior skin cancers and location (*p* = 0.04), history of prior skin cancers and histology (*p* = 0.04), and tumour thickness and age (*p* = 0.04) were found in the Hispanic group, but subsequent pairwise analyses for each category did not demonstrate significant associations. There was, however, a significant association between sex and histology (*p* = 0.004), and subsequent analysis showed that Hispanic men were more likely to have an aggressive mix (33.3%) than Hispanic women (11.3%) (*p* = 0.004). No other clinicopathologic features were found to be statistically significant.

## 4 DISCUSSION

Basal cell carcinoma is the most common malignancy in the United States, and yet knowledge of this disease among Hispanics remains limited. Throughout this study, our aim was to further investigate this understudied population, and help identify potential clinical gaps that may be important for this particularly unique subset of patients. Furthermore, it was also our goal to conduct this study among Hispanics, as they make up the largest proportion of minoritised patients in the US. Our findings add to the growing body of evidence that BCC may have distinct clinicopathologic characteristics among Hispanics, when compared to NHW. We found statistically significant differences amongst gender, anatomic location, and BCC pigmentation. Specifically, we found that when comparing Hispanic to NHW patients, they were predominately women, they had an increased incidence of head and neck BCC and demonstrated a higher incidence of pigmented BCC when compared to NHW. In concordance with at least three other studies,^10^, ^24^, ^25^ we found a significant gender disparity between Hispanic patients with BCC compared to NHW, whereby a higher proportion of Hispanics who had BCC tumours were women (68.4%), compared to men (31.6%). We hypothesise that the reasons for this gender difference are multifactorial, spanning a mix of biological, cultural, geographical, and socioeconomic factors. As others have suggested, it is possible there may be surveillance bias, due to heightened attentiveness and dermatologic follow‐up among Hispanic women,^10^, ^26^, ^27^ compared to Hispanic men. For example, in a study conducted by Perper et al., they demonstrated that greater proportions of Hispanic women presented with squamous cell carcinoma in situ compared to squamous cell carcinoma in Hispanic males, supporting the hypothesis that men may present for evaluation at later stages.^12^ Our study further supports this hypothesis, as Hispanic men had significantly higher rates of aggressive mix BCCs, as opposed to nonaggressive mix BCCs in Hispanic women. Overall, numerous studies have shown that Hispanics and other POC have lower awareness regarding skin cancer risk and sun protection behaviour.^13^, ^15^, ^16^ Interventions to help detect skin cancers and other BCC among POC may be complex, as POC have been reported to be less able to recognise skin cancers as well.^17^ Furthermore, many studies have documented that Hispanic patients are less likely to practice self‐skin examination.^15^, ^28^, ^29^, ^30^ Considering these factors, we believe that: greater efforts should be invested in improving overall awareness, access to care and outreach regarding BCC and other skin cancers, including improving sun protection behaviours and education on self‐skin examination, for patients of Hispanic ethnicity.

Regarding anatomic location, we found that the most common tumour site for BCC among our cohort of Hispanic patients was, by far, the head/neck region. The vast majority (90.1%) of BCC tumours among Hispanics were located at the head/neck, significantly higher than among NHW. This percentage is also higher than that reported by other studies.^10^, ^12^, ^31^ Chronic ultraviolet (UV) exposure in head/neck regions make this anatomical location more vulnerable to BCC.^32^ In particular, the risk in Hispanics may be further compounded by the fact that this population is less likely to engage in sun protective behaviours,^28^, ^33^, ^34^ despite being the racial group with the greatest occupational sun exposure.^33^ Additionally, Hispanics from Mexico have a lower sunburn threshold than Hispanics from several other regions, such as Central/South America and Cuba.^28^ With 75% of Hispanics in the Los Angeles area originating from Mexico,^35^ this may explain why the prevalence of head/neck BCC in our Hispanic patients was much greater than Hispanics in other studies, which were mostly conducted in the East Coast. Nonetheless, given that head and neck regions are considered high risk locations, this further highlights the need for early detection and treatment of BCC to avoid considerable consequences. Specifically, future interventions to specifically counsel Hispanic populations on the need for sun protection for the head/neck may be warranted. When comparing histology across racio‐ethnic groups, there was a higher prevalence of nodular histology in Hispanics compared to the NHW, though this did not meet statistical significance. While previous studies suggest that Hispanics may have less aggressive histological features of BCC,^10^, ^19^, ^36^ this is contradicted by the fact that Hispanics with BCC have worse clinical outcome.^10^, ^11^, ^31^, ^36^ Additionally, we also found 58% of BCC in Hispanics were pigmented as opposed to less than 16% in NHW. Clinically, pigmented basal cell carcinomas (PBCC) are more common in POC,^37^, ^38^ with studies estimating the prevalence of PBCC to be as high as 50%–65% among Hispanics^6^, ^25^, ^39^, ^40^ compared to only 5%–11% among NHW.^25^, ^41^, ^42^ This discrepancy in PBCC prevalence may in part be explained by surveillance bias. Pigmented lesions are far more recognisable, especially if located on cosmetically sensitive locations, and may motivate patients to seek care; thus, it is possible that surveillance bias may not only affect which POC will present with BCC, but also which histology types as well. Nevertheless, PBCC may be clinically challenging, as they may be mistaken for melanoma,^39^, ^43^ or even benign conditions, such as seborrhoeic keratosis or nevus sebaceous, and may also be difficult to judge in terms of response to treatment.^44^ Beyond histology, recent studies have identified differences between racio‐ethnic groups with BCC at the genetic level as well, with notable differences in alterations of cancer‐related genes between Caucasians and POC, as well as lower rates of UV‐related mutations among POC.^24^, ^45^, ^46^ Further studies of the underlying molecular and biological differences between BCC among different races will be critical going forward, to explore their clinical relevance and their potential for intervention among at risk populations.

Limitations to our study include its retrospective nature, lack of genomic correlatives and the use of a small study population restricted to a single institution. Future studies investigating the validity of our findings will need to be conducted amongst a larger patient population across multiple institutions. However, this study was conducted at one of the largest public hospitals in the United States, thus serving not only a highly diverse population, but also inclusive of those from lower socioeconomic backgrounds and potentially those with limited access to health care, compared to the general public. We believe this to be a unique and important strength of our study, as 49% of the population of the city of Los Angeles identify as Hispanic/Latino.^18^ As well, Hispanic ethnicity comprises diverse backgrounds, originating from a variety of regions and heritages. It is estimated that roughly 75% of the Hispanic population in Los Angeles originate from Mexico,^35^ which does distinguish our study population from other studies conducted among Hispanic populations in other regions of the USA or other countries as well.

In closing, we reinforce the presence of differences between BCC patients of Hispanic ethnicity compared to NHW, noting that BCCs among Hispanics predominantly are diagnosed among women, are located on the head/neck, with more frequent nodular and pigmented histology subtypes. In addition, Hispanic men in this study presented with more aggressive subtypes compared to Hispanic women and in particular, may be at higher risk. We hope that this study further influences better advocacy and attention as well as greater resources to support this under‐represented population, with a focus on improved sun protective behaviour, especially shielding the head and neck region, and skin cancer education for Hispanic populations. Furthermore, we hope that continued efforts to raise clinician awareness and improve the diagnosis and treatment of various skin conditions among POC should also be prioritised.

## CONFLICT OF INTEREST STATEMENT

GKI reports the following disclosures: Research support (institutional) from Regeneron, Array, Idera, Replimune, Xencor, InstilBio, Iovance, Pfizer, Checkmate Pharmaceuticals; Advisory Boards for Castle Biosciences, Sanofi, Replimune, Pfizer, Regeneron, Novartis, BMS. Speaker for Merck. EC, MAO, MH, SC, KM, AM, and LH have no conflicts of interest to declare.

## AUTHOR CONTRIBUTIONS

**Esther Choi**: Data curation (equal); formal analysis (equal); investigation (equal). **Martha A. Oberg**: Data curation (equal); formal analysis (equal); investigation (equal). **Maya Hijazi**: Data curation (equal); formal analysis (equal); investigation (equal); writing – review & editing (equal). **Luke Hall**: Formal analysis (equal). **Kimberly A. Miller**: Writing – review & editing (equal). **Arjun Mehta**: Conceptualisation (equal); writing – review & editing (equal). **Stephen Capone**: Writing – review & editing (equal). **Gino K. In**: Conceptualisation (equal); data curation, funding acquisition (equal); investigation. methodology (equal); project administration (equal); resources (equal); supervision (equal); visualisation (equal); writing – original draft (equal); writing – review & editing.

## ETHICS STATEMENT

This study was approved by the Institutional Review Board of the University of Southern California (IRB # HS‐16‐00840).

## Data Availability

The deidentified datasets generated during the current study are available from the corresponding author on reasonable request.
